# High-Grade Biofuel Synthesis from Paired Electrohydrogenation and Electrooxidation of Furfural Using Symmetric Ru/Reduced Graphene Oxide Electrodes

**DOI:** 10.1021/acsami.1c02231

**Published:** 2021-05-19

**Authors:** G. Bharath, Fawzi Banat

**Affiliations:** Department of Chemical Engineering, Khalifa University, P.O. Box 127788, Abu Dhabi 127788, United Arab Emirates

**Keywords:** furfural, 2-methylfuran, paired electrolyzer, electrocatalytic hydrogenation, electrocatalytic oxidation, sustainable liquid fuel

## Abstract

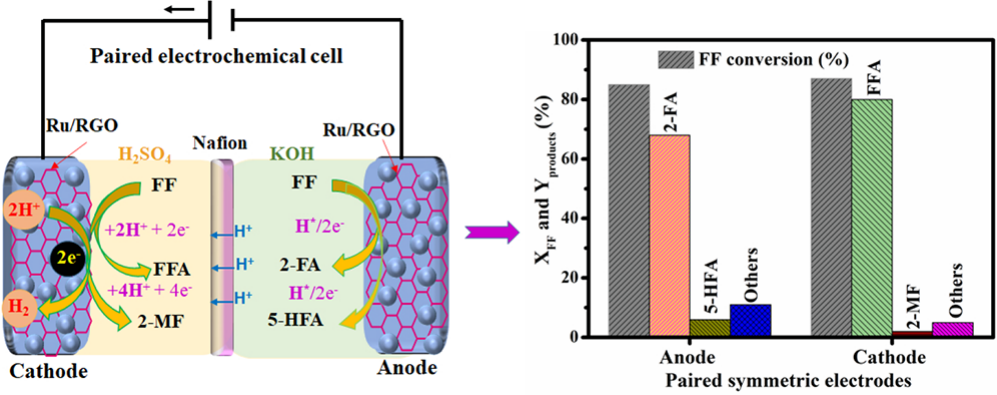

Electrochemical hydrogenation is a challenging technoeconomic process for sustainable liquid fuel production from biomass-derived compounds. In general, half-cell hydrogenation is paired with water oxidation to generate the low economic value of O_2_ at the anode. Herein, a new strategy for the rational design of Ru/reduced graphene oxide (Ru/RGO) nanocomposites through a cost-effective and straightforward microwave irradiation technique is reported for the first time. The Ru nanoparticles with an average size of 3.5 nm are well anchored into the RGO frameworks with attractive nanostructures to enhance the furfural’s paired electrohydrogenation (ECH) and electrooxidation (ECO) process to achieve high-grade biofuel. Furfural is used as a reactant with the paired electrolyzer to produce furfuryl alcohol and 2-methylfuran at the cathode side. Simultaneously, 2-furic acid and 5-hydroxyfuroic acid along with plenty of H^+^ and e^–^ are generated at the anode side. Most impressively, the paired electrolyzer induces an extraordinary ECH and ECO of furfural, with the desired production of 2-methylfuran (yield = 91% and faradic efficiency (FE) of 95%) at *X*_FF_ = 97%, outperforming the ECH half-cell reaction. The mechanisms of the half-cell reaction and paired cell reaction are discussed. Exquisite control of the reaction parameters, optimized strategies, and the yield of individual products are demonstrated. These results show that the Ru/RuO nanocomposite is a potential candidate for biofuel production in industrial sectors.

## 1 Introduction

Electrochemical conversion processes have attracted immense interest in sustainable liquid fuel production because they utilize electrical energy to transform biomass-derived compounds into high value-added products.^1−3^ In a typical electrolyzer cell, two oxidation and reduction half-reactions are involved in the conversion process. In particular, the active hydrogen (*H*_ads_) required for the electrohydrogenation (ECH) of bio-oil compounds at the cathode is mainly produced at the anode surface by water oxidation (2H_2_O → 4H^+^ + 4e^–^ + O_2_).^4^ Mostly, the ECH process is paired with water oxidation to produce a low economic value O_2_ at the anode surfaces.^5^ However, the ECO process is applied to bio-oil compound oxidation to generate value-added oxygenated products along with abundant H^+^ and e^–^ discharged at the anode surfaces.^6^ The discharged H^+^ and e^–^ effectively participate in the ECH reactions to enhance the hydrogenated products’ yield.^7^ In this context, a paired electrolyzer has been constructed for simultaneous bio-oil compound reduction and oxidation into high value-added chemicals. In this paired electrolyzer, the anodic products H^+^ and e^–^ were the inputs for the cathodic ECH of bio-oil compounds to minimize the conversion process’s energy demand.^8^ Recent efforts demonstrated the effective utilization of both anodic and cathodic sides for the simultaneous ECH and ECO of bio-oil compounds.^8,9^ In particular, the bio-oil compounds, such as furfural (FF) and 5-hydroxymethylfurfural (HMF), and phenolic compounds have been successfully converted into alcohols, fuels, and oxidative reactive products using paired electrolyzers.^5,9−14^

FF has recently been investigated as an essential intermediate precursor for producing various chemicals, including furfuryl alcohol (FFA), 2-methylfuran (2-MF), 5-hydroxy-2(5H)-furanone, 2-furoic acid (2-FA), maleic acid (MA), and 5-hydroxyfuroic acid (5-HFA), under paired electrolysis conditions. Recently, a Ni_2_P/nickel foam electrocatalyst system has been fabricated and employed as a bifunctional catalyst in the paired electrolyzer for the furfural oxidation and production of H_2_ with 100% Faradaic efficiencies.^6^ Furthermore, nanostructured transition-metal phosphides such as Cu_3_P/CFC and Ni_2_P/CFC have been studied as asymmetric electrodes in the paired electrolyzer for the simultaneous ECH and ECO of furfural at a controlled cathodic potential.^10^ It was observed that FF was successfully hydrogenated into FFA at the Cu_3_P/CFC electrode, while the Ni_2_P/CFC electrode could potentially be involved in FF oxygenation into 2-FA under the same experimental conditions. In particular, a paired electrode system was effectively utilized for the synthesis of 2-FA, 5-HFA, 2-MF, and FFA from FF under various operating conditions.^10^ Among them, 2-MF has a high octane number and can be directly used in gasoline blends for producing diesel/jet fuel hydrocarbons.^15^ FF is a potential candidate for 2-MF production.^16^ The selective production of high-yield 2-MF via the ECH process has rarely been studied.^5,17−21^ 2-MF can be formed with a high potential (−0.55 to −1.5 V) and/or strongly acidic solutions, and the highest reported 2-MF yields (20–75%) from the ECH process used 500 mM H_2_SO_4_ as the electrolyte over copper, lead, and Pt/C cathodes.^19,20,22^ Hence, high-performance and low-cost electrode materials are vital for producing high-yield 2-MF under paired electrolyzer conditions.

Herein, we report the rational design of Ru/reduced graphene oxide (Ru/RGO) nanocomposites with numerous porous networks through a cost-effective and straightforward microwave irradiation process and employed these as the paired electrolyzer for selective 2-MF production. The morphological and structural investigations of the Ru/RGO nanocomposite were studied by HR-TEM, SEM, XRD, Raman spectroscopy, N_2_ sorption isotherms, and XPS analyses. The Ru/RGO nanocomposite catalytic activities were studied by cyclic voltammetry (CV) in the presence and absence of FF under various electrolytic conditions. At first, the half-cell reactions of ECH and ECO of FF were conducted separately to determine the FF conversion performance (*X*_FF_) and product yields over the Ru/RGO electrode. The effects of FF concentration, applied cell potential, and electrolyte concentration vs *X*_FF_ and product yields were examined. Based on the optimization, a paired electrochemical cell was constructed from Ru/RGO as common electrodes, and the cell potential was negatively controlled by −1.25 V vs Ag/AgCl. The Ru/RGO||Ru/RGO electrolyzer displayed a higher 2-MF yield of 91% at a higher *X*_FF_ = 97% on the Ru/RGO cathode surfaces, outperforming the half-cell electrolyzer. Therefore, we believe that the present fabrication strategy can be translated for the rational design of several nanocomposites and effective catalysts to convert FF into value-added alcohols, fuels, and industrial chemicals.

## 2 Experimental Section

### 2.1 Synthesis of the Ru/RGO Nanocomposite

The modified Hummers’ method was used for the preparation of GO through harsh oxidation of graphite flakes, as demonstrated elsewhere.^23−25^ The Ru/RGO nanocomposite was synthesized using the following detailed procedure: 100 mg of GO was dispersed in 50 mL of deionized water (2 mg mL^–1^) under sonication for 30 min. Then, 100 mM ruthenium(III) chloride hydrate (RuCl_3_·*x*H_2_O, 99.98%) was dissolved into a 50 mL GO suspension under magnetic stirring for 15 min. In this stage, Ru(III) ions were electrostatically anchored with the oxygenated functional groups on the GO surfaces to form a Ru(III)/GO mixture. Meanwhile, 50 mM NaBH_4_ (99%) as a reducing agent was dissolved in 10 mL of deionized water, which was added dropwise into the above Ru(III)/GO mixture. Primary Ru° ions were formed on the GO surfaces, while GO was chemically reduced into RGO, resulting in the formation of a Ru°/RGO suspension. Furthermore, the complete suspension was kept in a microwave oven for 30 s to allow the secondary growth of the metallic Ru nuclei on the RGO surfaces. The Ru/RGO precipitate was separated and washed with deionized water followed by ethanol several times to remove the chemical impurities. Finally, the obtained Ru/RGO nanocomposite powder was dried in a vacuum oven at 70 °C overnight. For comparison, pure Ru NPs were synthesized using a similar procedure without GO. Additionally, GO was chemically reduced into RGO under microwave irradiation for 30 s, using 50 mM NaBH_4_ as the reducing agent.

### 2.2 Morphological and Structural Characterization

The morphological analysis of the as-obtained nanocomposite was performed using a scanning electron microscope (SEM; JEOL JSM-7610F FEG-SEM) with EDS and HR-TEM (Titan TEM 300 kV) with the fast Fourier transform pattern. A PANalytical Empyrean at 40 kV and 40 mA with Cu Kα radiation and a Witec Alpha 300 Raman spectrometer (laser wavelength of 532 nm) were used to investigate the sample crystallinity and structure. The specific surface area and pore size distributions of the as-obtained nanocomposites were determined using Quantachrome Autosorb 06 at −196 °C under N_2_. The sample was degassed at 250 °C for 4 h to remove atmospheric impurities. The XPS ESCALab MKII was used to analyze the chemical composition and chemical states of the Ru/RGO nanocomposite.

### 2.3 Electrohydrogenation of Furfural

Electrochemical performances of pure Ru and the Ru/RGO nanocomposite were evaluated in a typical three-electrode configuration using a Biologic VMP-300 electrochemical workstation. A certain amount of sample was drop cast onto a carbon sheet and used as the working electrode, and Ag/AgCl and Pt sheets were used as the reference and counter electrodes, respectively. The CVs were recorded at a fixed scan rate of 20 mV s^–1^ within a potential window of 0 to −1.6 V vs Ag/AgCl in 1 M H_2_SO_4_ as the electrolyte solution, while 20 mM furfural was added during the CV measurements under slight magnetic stirring of 20 rpm. H_2_SO_4_ was employed as the electrolyte for the selective production of 2-MF. The CV was recorded without the addition of furfural over the Ru/RGO-based electrode. Additionally, the effect of CV characteristics vs various furfural concentrations (5, 10, and 20 mM) was recorded at 20 mV s^–1^ with a potential window of 0 to −1.6 V vs Ag/AgCl in 1 M H_2_SO_4_ as the electrolyte solution over the Ru/RGO-based electrode. Based on CV studies, ECH of furfural was carried out in a two-compartment gas-tight H-type cell separated by a proton exchange membrane (Nafion 117) over a Ru/RGO-based electrode at an applied negative potential of −1.25 V vs Ag/AgCl in 1 M H_2_SO_4_ electrolyte solution for 120 min (optimized ECH time). Furthermore, various furfural concentrations (5–30 mM), electrolyte concentrations (0.1–2.0 M H_2_SO_4_), and applied potential (−0.8 to −1.4 V vs Ag/AgCl) vs ECH of furfural were examined. The liquid products were analyzed using an Agilent Gas chromatography mass spectrometry (GC/MS) system (Agilent 7890B GC and 5977A MSD) equipped with flame ionization detection (GC-FID). The furfural conversion efficiency (*X*), ECH product yield (*Y*), selectivity (*S*), and faradic efficiency (FE) were estimated using eqs 1–4
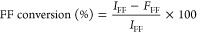
1
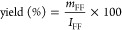
2
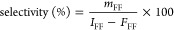
3
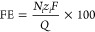
4where *F*_FF_ is the number of moles of furfural consumed, *I*_FF_ is the number of moles of initial furfural, *m*_FF_ is the number of moles of the obtained products, *F* is the Faraday constant (*F* = 96 485 C/mol), *z_i_* is the number of electrons transferred per molecule of product, *N_i_* is the number of moles of product *i*, and *Q* is the total charge passed in the circuit.

### 2.4 Electrooxidation of Furfural

In a typical three-electrode configuration setup, the as-obtained catalyst-coated carbon sheets were used as the working electrode, and Ag/AgCl and Pt sheets were used as the reference and counter electrodes, respectively. The CV curves were recorded on the as-obtained catalysts in aqueous KOH (1 M) in the absence and presence of furfural in the operating voltage window of 0 to +1.4 V vs Ag/AgCl at a fixed scan rate of 20 mV s^–1^ under slight magnetic stirring of 20 rpm. Notably, KOH was used as an electrolyte in an anodic chamber for better conductivity and for the selective production of 2-FA and 5-HFA. The effect of furfural concentrations (2, 10, and 20 mM) was determined through CV measurements with an operating potential window of 0 to +1.4 V vs Ag/AgCl at a fixed scan rate of 20 mV s^–1^. Based on the optimized conditions, ECO of furfural was carried out in a two-compartment gas-tight H-type cell separated by a proton exchange membrane (Nafion 117) at 1.0 V in 1 M KOH electrolyte solution for 120 min as the optimized ECO time. Moreover, the ECO of furfural and product yields vs various furfural concentrations (5–30 mM), electrolyte concentrations (0.1–2.0 M KOH), and applied potential (0.8–1.4 V vs Ag/AgCl) were studied over the Ru/RGO electrode. The liquid products were analyzed using an Agilent gas chromatography mass spectrometry (GC/MS) system (Agilent 7890B GC and 5977A MSD) equipped with GC-FID. The furfural conversion efficiency (*X*), ECO product yield (*Y*), ECO selectivity (*S*), and faradic efficiency (FE) were estimated using eqs 1–4.

### 2.5 Paired Electrolysis Cell

In the paired electrolyzer, a Ru/RGO-coated carbon sheet was used as the common electrode for both the anodic oxidation and cathodic hydrogenation of furfural while controlling the cathode potential at −1.25 V vs Ag/AgCl. The anodic chamber was filled with 1 M KOH, while the cathodic chamber was filled with 2.0 M H_2_SO_4_, which was separated by a proton exchange membrane. After the reaction, the liquid products from the catholyte and the anolyte were collected separately and analyzed by GCMS (Agilent 7890B GC and 5977A MSD) equipped with GC-FID.

## 3 Results and Discussion

### 3.1 Physicochemical Properties

**Figure 1:**
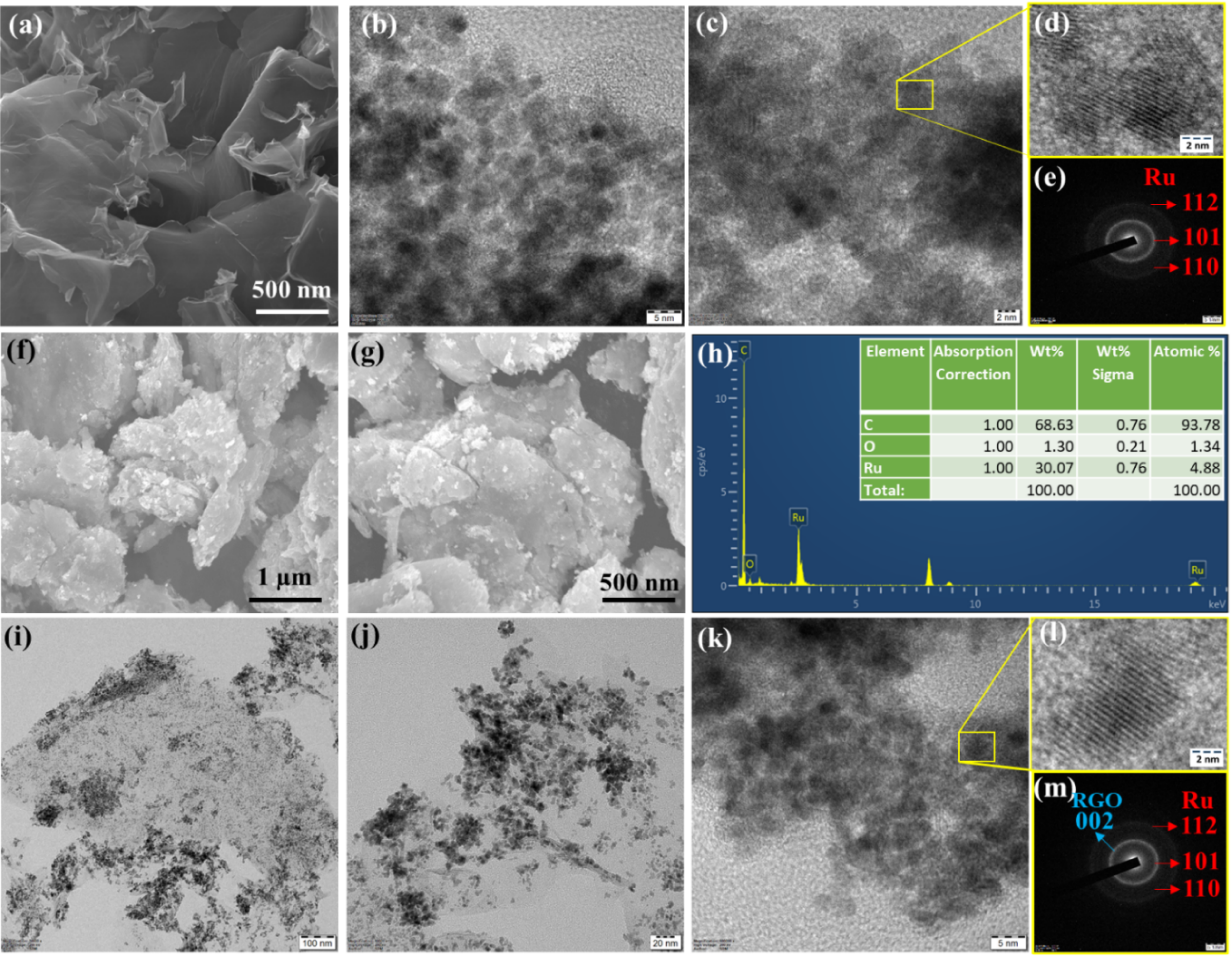
(a) FE-SEM image of RGO, (b–d) HR-TEM images of Ru NPs, (e) SAED pattern of Ru NPs, (f and g) FE-SEM images of Ru/RGO nanocomposites, (h) EDS analysis of Ru/RGO nanocomposites, (i and j) low magnification TEM images of Ru/RGO nanocomposites, (k and l) high-resolution TEM (HR-TEM) images of Ru/RGO nanocomposites, and (m) SAED pattern of the Ru/RGO nanocomposites.

The Ru/RGO nanocomposite was rationally designed using the simple and cost-effective microwave process in the presence of NaBH_4_ as a reducing agent. The SEM image of pristine RGO in Figure 1a clearly shows ultrathin, smooth, and transparent two-dimensional (2D) nanosheets. Note that microwave irradiation afforded an effective, low-cost technique to synthesize defect-free RGO nanosheets, enhancing the electronic and ionic conductivity during the electrochemical process.^26,27^ The microwave irradiation process was also used to synthesize ultrasmall Ru NPs with spherical Ru NPs in the presence of NaBH_4_. Initially, Ru^3+^ ions were completely reduced into Ru° nuclei by NaBH_4_, while the formed Ru° nuclei underwent secondary growth under a microwave irradiation process.^16^ The rapid heating rate at a shorter reaction time promoted highly crystalline ultrasmall Ru NP formation and avoided aggregation.

The HR-TEM images in Figure 1b,c show the evidence for the formation of ultrasmall Ru NPs under the microwave irradiation process. The synthesized Ru NPs had a sphere-like morphology with particle sizes in the range of 3–5 nm, which was homogeneously distributed throughout the TEM micrographs. The HR-TEM image in Figure 1d shows the lattice plane in the individual Ru NPs, demonstrating a *d*-spacing of 0.27 nm, which corresponded to the (101) facets of hexagonal close-packed (hcp) Ru.^16^ The SAED pattern in Figure 1e displays clear diffraction rings of (110), (101), and (112) corresponding to polycrystalline hcp Ru, indicating pure Ru NP formation under microwave irradiation.^28^ The FE-SEM images in Figure 1f,g show the morphology of the as-prepared Ru/RGO nanocomposite. The magnified FE-SEM image in Figure 1g shows that the smaller diameter Ru NPs were successfully anchored onto the RGO framework. Figure 1h displays the EDS spectra of the Ru/RGO nanocomposite, which consisted of Ru, C, and O with atomic percentages of 93.78%, 1.34%, and 4.88%, respectively, while no other impurities are observed.

TEM analysis further proves that ultrasmall Ru NPs were homogeneously anchored onto the RGO nanosheets (Figure 1I,j). Ru NPs with an average size of 3 nm were observed on the RGO nanosheet surfaces, as displayed in Figure 1k. The Ru particle size distribution in the Ru/RGO nanocomposite was relatively narrower than that in the pure Ru NPs (4 nm). In particular, RGO sheets possessed Ru° nuclei anchoring sites, which controlled the secondary Ru° nucleation growth under the microwave process, resulting in the formation of ultrasmall Ru NPs.^27,29,30^ A lattice fringe with a *d*-spacing of 0.241 nm was attributed to the (101) facets of individual hcp Ru NPs, as clearly observed in Figure 1.^28^ Furthermore, as found from the SAED pattern, RGO exhibited a (002) diffraction ring corresponding to the graphitic C=C structure, while clear diffraction rings of (110), (101), and (112) corresponded to the single-crystalline hcp Ru, thus confirming the dual RGO and Ru NP phase formation in the Ru/RGO nanocomposites.^28,31^

**Figure 2:**
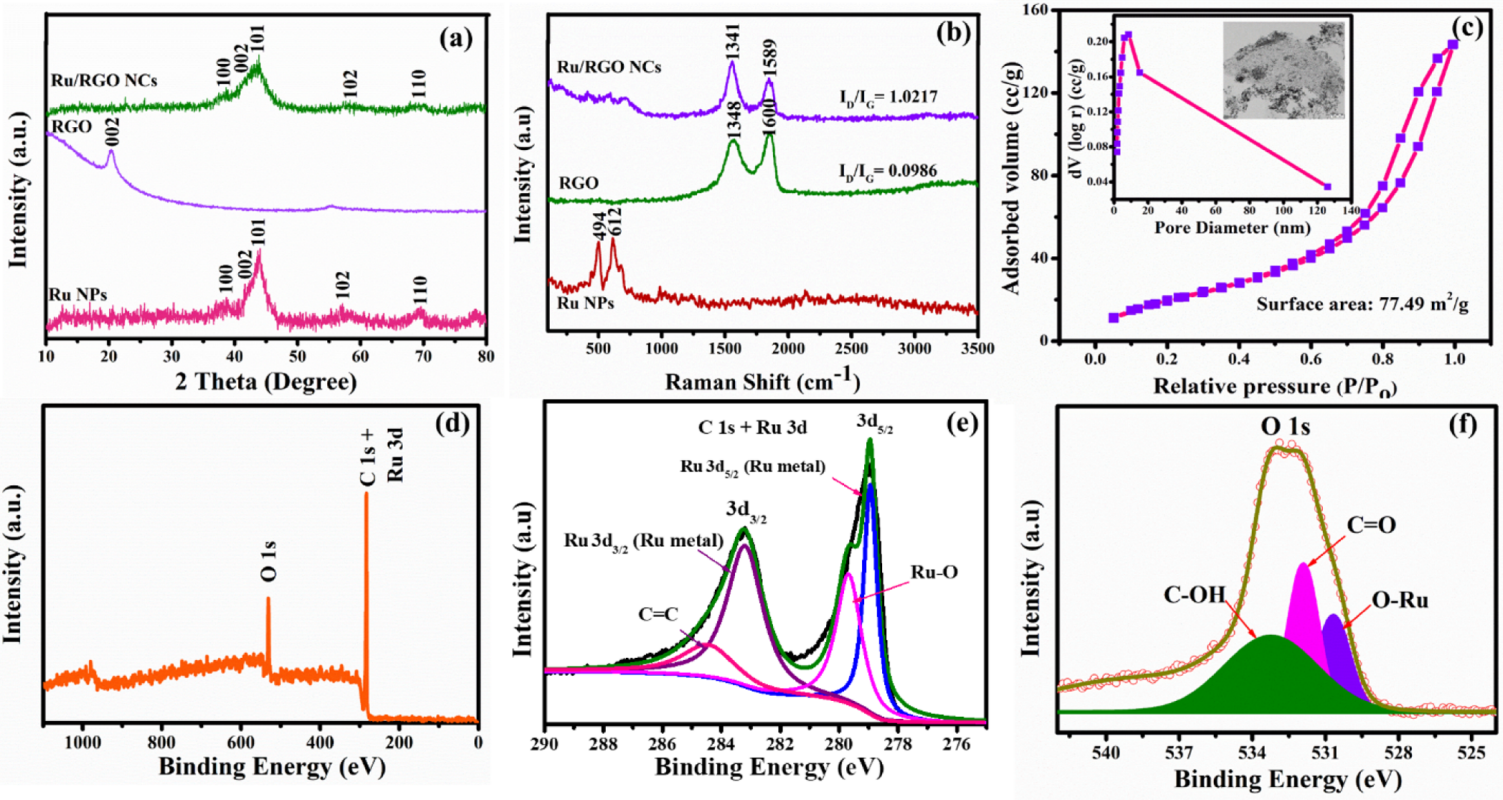
(a) XRD patterns of Ru NPs, RGO, and Ru/RGO NCs, (b) Raman analysis of the Ru NPs, RGO, and Ru/RGO NCs, (c) BET N_2_ sorption isotherms plots of the Ru/RGO NCs (the inset shows the corresponding BJH pore size distributions curve), and (d) the survey XPS spectrum of the Ru/RGO NCs. The high-resolution XPS of (e) C 1s + Ru 3d and (f) O 1s.

The crystallographic phases and crystallite sizes of the pure Ru NPs, RGO, and Ru/RGO nanocomposite were determined by X-ray diffraction analysis, and the results are displayed in Figure 2a. The pure Ru NPs exhibited the crystal planes of (100), (002), (101), (102), and (110) at diffraction angles of 2θ = 38.5°, 42.9°, 44.1°, 58.1°, and 69.3°, respectively. This indicated the successful hcp Ru crystal formation (ICDD-JCPDS card no. 06-0663) under the microwave irradiation process.^16,32,33^ In the case of RGO, the XRD pattern in Figure 2a shows an intense peak at 25.2° corresponding to the graphitic C=C carbon structure (002) facet (ICDD-JCPDS card no. 41-1487). This demonstrated that the microwave irradiation process was effectively involved in the GO reduction process and graphitic carbon structure restoration in the presence of NaBH_4_. In the case of Ru/RGO nanocomposite, the XRD pattern exhibited crystal planes of (100), (002), (101), (102), and (110) at diffraction angles of 2θ = 38.6°, 42.5°, 44.0°, 58.2°, and 69.4°, respectively, while the RGO crystal plane of (002) disappeared due to successful high-intensity hcp Ru crystal formation on RGO surfaces.^29,31^ The Scherrer equation was used to determine the average Ru NPs and Ru/RGO nanocomposite crystallite sizes. The crystallite sizes of 4.5 and 3 nm corresponded to Ru NPs and Ru/RGO nanocomposites, respectively. Additionally, the high-intensity diffraction crystal plane of (101) was used to determine the pure Ru NPs and Ru/RGO nanocomposite lattice constants using the Rietveld refinement: *a* = 0.2217 nm and *c* = 0.3517 nm for the pure hcp Ru lattice and *a* = 0.2117 nm and *c* = 3411 nm for the hcp Ru lattice in the Ru/RGO composite.^16,27,32^ The decreased Ru/RGO nanocomposite lattice constant indicated the formation of smaller Ru NPs on the RGO surfaces under microwave irradiation, which was consistent with the FE-SEM and HR-TEM analyses. Hence, controlling the Ru NP crystallite sizes on the RGO surfaces effectively increased the electrocatalytic activity for the ECH and electrooxidation (ECO) of furfural into value-added chemicals. Furthermore, Raman analysis is a powerful technique for understanding the structural information of carbon-based nanomaterials. Figure 2b depicts the Raman spectra of pure Ru NPs, RGO, and the Ru/RGO nanocomposite. The Raman spectra of Ru NPs in Figure 2b shows two intense peaks at 494 and 612 cm^–1^ corresponding to the Ru–O A_1g_ and E_g_ orbitals, which indicated Ru NP atmospheric surface oxidation.^34^ Microwave-assisted chemical RGO reduction exhibited two intense prominent peaks at 1348 and 1600 cm^–1^ corresponding to the defect D band (sp^3^) and the graphitic G band (sp^2^), respectively.^29,34^ In general, the D band arose due to the presence of oxygen moieties on RGO surfaces, and the G band is due to the in-plane sp^2^ (C=C) aromatic carbon structure vibration existing on the as-prepared RGO under microwave irradiation.^30^

The RGO defect level was estimated as 0.986 through the *I*_D_/*I*_G_ intensity ratio, which was lower than that of RGO prepared from other chemical reduction processes.^29^ Microwave irradiation is a favorable reduction process for preparing smooth, high-quality, and defect-free RGO, which potentially enhanced electrical transport properties for electrochemical applications. In addition, the Raman spectra of Ru/RGO exhibited two strong peaks at 1341 and 1589 cm^–1^ corresponding to the D and G bands, respectively, while the *I*_D_/*I*_G_ ratio was estimated to be 1.0217. An increased intensity ratio with slight shits in the D and G bands were observed for the Ru/RGO nanocomposite compared to unmodified RGO. This was due to the disorderness in the structure of the nanocomposite, which was induced by the incorporation of Ru NPs on the RGO sheets under the microwave field.^29,35^ Nitrogen adsorption–desorption measurements were employed to estimate the specific surface area and pore size distribution of the Ru/RGO nanocomposite, as depicted in Figure 2c. The Ru/RGO nanocomposite isotherms were categorized as type III isotherms in the IUPAC classification terms at *P*/*P*_0_ = 0.5–0.9, demonstrating that the mesopore’s successful formation throughout the Ru/RGO nanocomposite during the controlled microwave irradiation process.^30^ The Ru/RGO nanocomposite possessed a higher specific surface area of 185 m^2^ g^–1^ and a total pore volume of 0.22 cm^3^ g^–1^. In particular, the smaller Ru NPs prevented RGO nanosheet agglomeration, which maintained the Ru/RGO nanocomposite specific surface area. Thus, the higher Ru/RGO nanocomposite pore volume and surface area provided larger available electrocatalytic reactive sites for FF conversion. XPS was further employed to analyze the Ru/RGO nanocomposite surface composition and chemical state, as displayed in Figure 2d–f. The Ru/RGO nanocomposite survey spectra in Figure 2d showed that the sample consisted of C 1s + Ru 3d and O 1s, while no other elements were observed. The deconvoluted high-resolution C 1s + Ru 3d spectra in Figure 2e were clearly distinguished between the overlapping C 1s and Ru 3d peaks.^16,32^ The deconvoluted Ru 3d_5/2_ and Ru 3d_3/2_ spin–orbit splitting was determined at binding energies of 279.07 and 283.67 eV, respectively, demonstrating the presence of the Ru metallic state of Ru, while a deconvoluted RuO_2_ 3d_5/2_ peak was found at 279.95 eV, suggesting the oxidation of the Ru surface under atmospheric conditions.^16^ The additional deconvoluted peak located at 284.2 eV was ascribed to the C–C/C=C in aromatic rings, which confirmed the presence of RGO in the Ru/RGO nanocomposite. Moreover, the deconvoluted O 1s XPS spectra in Figure 2e exhibited three intense peaks, namely, O–Ru at 530.6 eV was attributed to Ru surface oxidation, C=O at 531.9 eV was ascribed to the remaining oxygenated elements on the RGO surface, and C–OH at 533.4 eV was related to the hydroxyl groups present on the Ru/RGO nanocomposite.^5,16,29^ The content of the remaining hydroxyl and carbonyl functional groups improved the wettability of Ru/RGO, which was a significant parameter influencing the electrochemical activity toward furfural conversion.

### 3.2 Cathodic Furfural Hydrogenation

**Figure 3:**
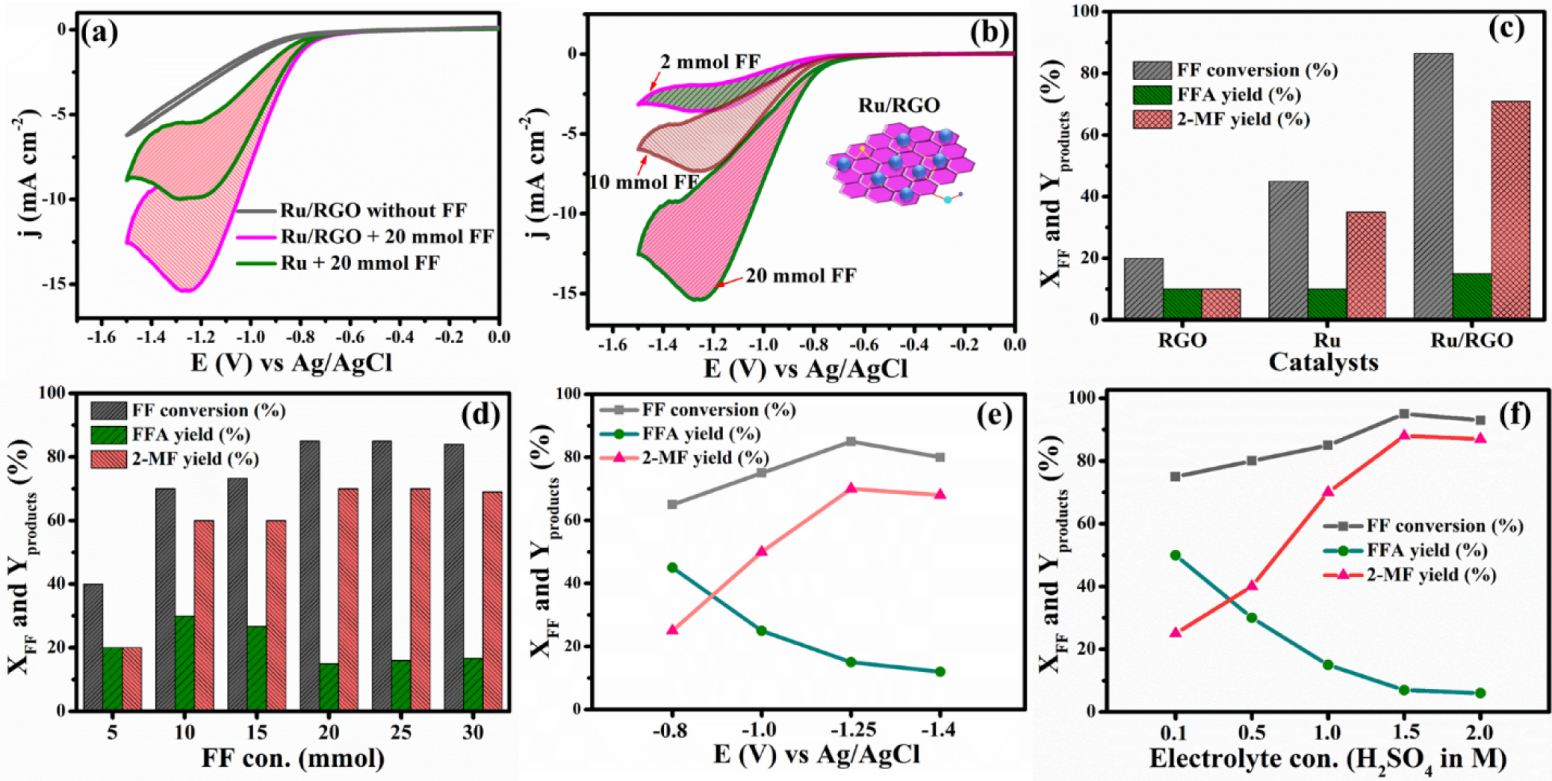
(a) CV characteristics of pure Ru NPs, Ru/RGO (absence and presence of FF) electrodes in 20 mmol FF and 1 M H_2_SO_4_ with a scan rate of 20 mV s^–1^ (not iR-compensated). (b) CV characteristics of Ru/RGO electrodes in 2, 10, and 20 mmol FF and 1 M H_2_SO_4_ with a scan rate of 20 mV s^–1^. (c) ECH of FF and yield of FFA and 2-MF over RGO, Ru, and Ru/RGO electrodes. Reaction conditions: 20 mmol FF, electrolyte 1 M H_2_SO_4_, optimum reaction time of 120 min, and potential of −1.25 V vs Ag/AgCl. (d) Various FF concentrations (5–30 mmol) vs ECH of FF and yield of FFA and 2-MF over Ru/RGO electrodes. Reaction conditions: electrolyte 1 M H_2_SO_4_, optimum reaction time of 120 min, and potential of −1.25 V vs Ag/AgCl. (e) Various applied potentials (−0.8 to −1.4 V) vs Ag/AgCl of FF and yield of FFA and 2-MF over Ru/RGO electrodes. Reaction conditions: 20 mmol FF, electrolyte 1 M H_2_SO_4_, and optimum reaction time of 120 min. (f) Various electrolyte concentrations (0.1–2.0 M H_2_SO_4_) vs ECH of FF and yield of FFA and 2-MF over Ru/RGO electrodes. Reaction conditions: 20 mmol FF, applied potential of −1.25 V vs Ag/AgCl, and optimum reaction time of 120 min. The electrochemical H-cell was used for conducting these experiments.

To attempt the hydrogenation of FF to FFA and 2-MF over Ru and Ru/RGO cathodes, CV was performed at a scan rate of 20 mV s^–1^ in 1 M H_2_SO_4_ as the electrolyte with and without the presence of FF. Figure 3a shows the CV behavior with and without 20 mM FF over Ru and Ru/RGO-modified carbon sheet cathodes. As revealed by the CV responses, the Ru electrode showed an obvious reduction peak current of FF at approximately −1.3 V in the presence of 20 mmol FF due to the cathodic reduction of FF to form FFA and 2-MF. Interestingly, the CV plot of Ru/RGO shows a larger cathodic peak current than that of Ru, with a lower overpotential shift to −1.25 V. The higher current density and the lower overpotential indicated that Ru/RGO is more active for the hydrogenation of FF compounds. The high-density mesopores of metallic Ru serve as active electrocatalytic sites, while RGO exposes wettability for the adsorption of FF molecules via electrostatic interactions, thereby providing a highly accessible surface area for ionic conductivity, which enhances the overall electrocatalytic reduction of FF at a lower overpotential. Additionally, a bare CV experiment was conducted in the absence of FF over the Ru/RGO electrode at 20 mV s^–1^ in 1 M H_2_SO_4_ electrolyte, which exhibited a relatively insignificant cathodic current due to a competing reaction such as the hydrogen evolution reaction (HER). To examine the electrochemically active surface area (ECSA) of the as-obtained electrodes, the electrochemical double-layer capacitance (*C*_dl_) was measured and is illustrated in Figure S1. The *C*_dl_ of the Ru and Ru/RGO electrodes were examined using CV measurements in a non-Faradic province using 1 M H_2_SO_4_ medium at different sweep rates from 10 to 100 mV s^–1^. The value of *C*_dl_ is usually calculated from the slope of the curve. The Ru/RGO electrode showed the highest *C*_dl_ value of ∼80.3 mF cm^–2^, which was significantly higher than that of the Ru electrode (22.1 mF cm^–2^). This clearly indicated that the Ru/RGO electrode possesses a large ECSA toward the electrochemical conversion of furfural. Figure 3b shows the influence of the FF initial concentration in the range of 2–20 mmol on the electroreduction of FF over the Ru/RGO electrode at 20 mV s^–1^ in a 1 M H_2_SO_4_ electrolyte. The cathodic peak currents increased with an increase in FF concentrations. This result indicated that the cathodic reduction currents were directly proportional to the FF concentrations under the same experimental conditions. The reduction of high concentrations of FF becomes easier on the Ru/RGO electrode due to the larger available binding sites adsorbing many FF molecules as well as favorable electron transfer from the electrode into catalytic sites of Ru via π–π conjugation of RGO. Despite the advantages of CV studies, the ECH of FF was conducted using a controlled potential of −1.25 V in 1 M H_2_SO_4_ as an electrolyte. In particular, the as-prepared Ru, RGO, and RU/RGO were used as common electrodes for fabricating ECH cells, and the anodic and cathodic chambers were separated with proton exchange membranes. The ECH of FF and product yields over various electrodes were determined in terms of FF conversion efficiency (*X*), yield (*Y*), selectivity (*S*), and faradic efficiency (FE). The main FF hydrogenation products were FFA and 2-MF over Ru-, RGO-, and Ru/RGO-based ECH electrodes, as displayed in Figure 3c. The results demonstrated that 20% of the FF was converted into 10% FFA yield and 10% 2-MF yield over microwave-synthesized RGO nanosheets with a carbon balance of 100%. Overall, the ECH of FF to FFA and 2-MF is an e^–^/FF = 2 and e^–^/FF = 4 process, respectively.^10,19^ Moreover, the FEs of FFA and 2-MF were 48% and 49%, respectively, at *X*_FF_ = 10%. Furthermore, a metallic Ru electrode exhibited product yields of 10.5% for FFA and 39% for 2-MF at *X*_FF_ = 51% with a carbon balance of 97%. Notably, a 1.5% yield of no traceable intermediates was observed by GC-MS and HPLC. The maximum 2-MF FE was estimated as 68%, indicating that the active Ru NP catalytic sites promoted the e^–^/FF = 4 process toward 2-MF formation.^19^ Interestingly, the FFA yield increased from 10.5% to 16%, and the 2-MF yield increased from 39% to 70.5% at *X*_FF_ = 86.5% with a carbon balance of 100% over Ru/RGO-based electrodes. The targeted 2-MF product determined a higher FE of 88% than the FEs of RGO (FE = 49%). In addition, Ru (FE = 68%) over RGO is an effective material for the adsorption of FF molecules, bringing FF near the catalytic sites for ECH of FF into FFA and 2-MF. Therefore, the Ru/RGO-based electrode showed superior electrocatalytic performance for the ECH of FF in the selective production of 2-MF along with FFA. Furthermore, the effect of FF concentration, various ECH cell potentials, and electrolyte concentration were determined over Ru/RGO-based electrodes, where the ECH of FF and yield of the hydrogenated products were determined by GC-MS and HPLC. Figure 3d depicts the effect of FF concentration vs *X*_FF_ and product yields of FFA (*Y*_FFA_) and 2-MF (*Y*_2-MF_) at −1.25 V vs Ag/AgCl in 1 M H_2_SO_4_ for 120 min over Ru/RGO-based electrodes. At a 5 mmol FF concentration, 20% FFA yield and 20% 2-MF yield were obtained at *X*_FF_ = 40% over Ru/RGO electrodes. Furthermore, the product yields rapidly increased with increasing FF concentration from 5 to 20 mmol and reached the maximum yields of 16% and 70.5% for FFA and 2-MF, respectively, at *X*_FF_ = 86.5% with a carbon balance of 98%. Eventually, a further increase in the FF concentration to 30 mmol did not improve the *X*_FF_ and the FFA and 2-MF yield due to the limited availability of catalytic sites for the ECH of FF in the area of the Ru/RGO electrode. However, we may increase the FF conversion efficiency and product yields by increasing the electrode area, which will be studied in future work. The effect of the applied potential (−0.8, −1.0, −1.25, and −1.4 V) on the ECH of FF in the 1 M H_2_SO_4_ electrolyte was investigated over the Ru/RGO electrode. The *X*_FF_ and yield of the hydrogenated products were tuned by varying the cathode potential, as depicted in Figure 3e. At a cathode potential of −0.8 V, a 45% FFA yield and 21.5% 2-MF yield at *X*_FF_ = 67% with a carbon balance of 99.5% was achieved. A lower cathode cell potential led to the e^–^/FF = 2 process, which was involved in FFA formation over the Ru/RGO electrode surfaces.^10,19^ Furthermore, the 2-MF yield rapidly increased as the cathodic cell potential was extended from −0.8 to −1.2 V vs Ag/AgCl and achieved the maximum yields of 70.5% and 16%, respectively, at *X*_FF_ = 86.5% with a carbon balance of 98%. As the cathode potential was increased to −1.4 V vs Ag/AgCl, the 2-MF and FFA yields decreased to 68% and 12% at *X*_FF_ = 80% due to the HER competition that occurred at a higher cathodic potential of −1.4 V vs Ag/AgCl, decreasing the ECH performance.^10^ Hence, the result of this investigation clearly demonstrated that −1.25 V vs Ag/AgCl is an optimized ECH cell potential for the selective conversion of FF into 2-MF. In addition, a strong acidic electrolyte condition was required for the selective production of 2-MF, with a higher FF conversion efficiency under optimized ECH conditions of −1.25 V vs Ag/AgCl as the applied cathodic potential, 120 min as the reaction time, and 20 mmol as the FF concentration.

**Figure 4:**
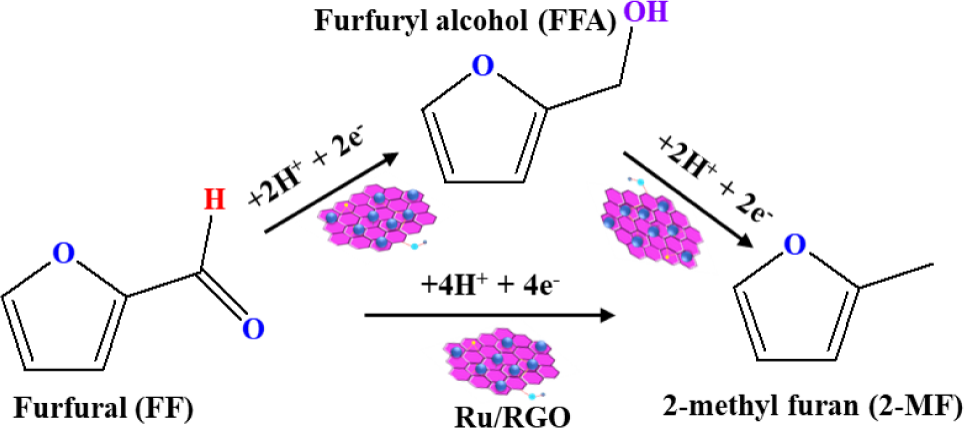
Plausible reaction scheme for ECH of FF into FFA and 2-MF over the Ru/RGO electrode.

The H_2_SO_4_ electrolyte concentration played a vital role in product yields and conversion of FF. When the H_2_SO_4_ concentration increases from 0.1 to 1.5 M in the cathodic chamber, the *X*_FF_ increased from 73% to 95%, and the 2-MF yield increased from 50% to 88%, with a maximum FE of 93%, while the FFA yield decreased from 23% to 7%, as displayed in Figure 3f. FFA production was high at 0.1 M H_2_SO_4_ concentration due to the e^–^/FF = 2 process dominating the e^–^/FF = 4 processes for 2-MF production in the cathodic chamber.^10^ However, a higher H_2_SO_4_ concentration (1.5 M) enhanced the e^–^/FF = 4 process, resulting in a higher 2-MF yield (*Y*_2-MF_ = 88%) compared with the lower acidic conditions.^19^ The two FF hydrogenation reactions occurred over the Ru/RGO cathode, and the overall FF hydrogenation into FFA and 2-MF occurred under acidic conditions, as depicted in Figure 4. Further increasing the H_2_SO_4_ electrolyte concentration to 2.0 M caused the 2-MF (87%) and FFA (6%) yields along with *X*_FF_ (93%) to slightly drop due to the desorption of more *H*_ads_ over the electrode surface, which enhanced the hydrogen evolution in the cathodic chamber.^5,10^ It is recommended to conduct the ECH of FF under the optimized conditions, including the desired half-cell voltage of −1.25 V, 20 mmol FF concentration, 1.5 M H_2_SO_4_ as the electrolyte solution, and 120 min as the electrolysis reaction time over Ru/RGO paired electrodes.

### 3.3 ECO of Furfural

**Figure 5:**
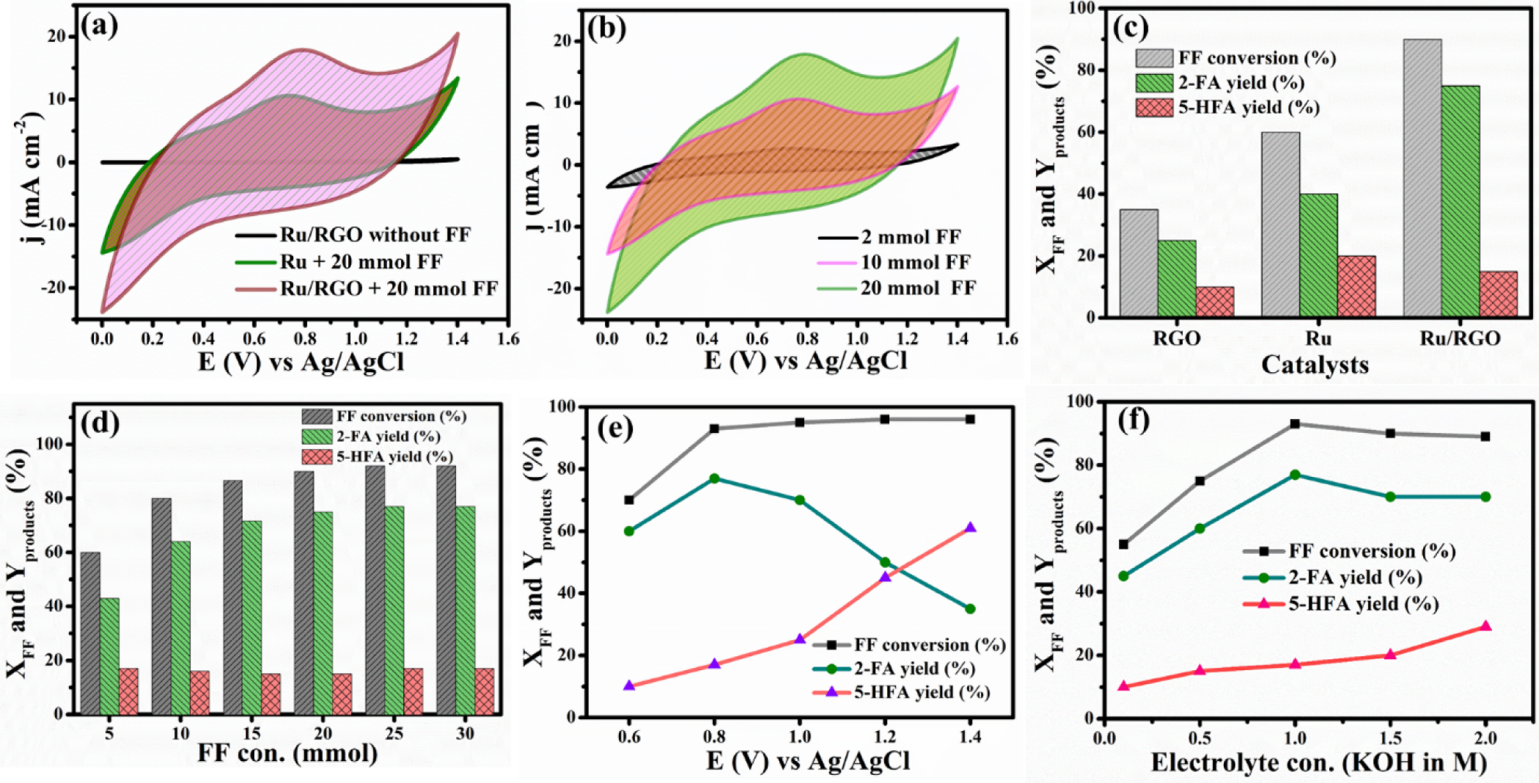
(a) CV characteristics of Ru, Ru/RGO, and bare (Ru/RGO without FF) electrodes in 20 mmol FF and 1 M aqueous KOH as the electrolyte with a scan rate of 20 mV s^–1^ (not iR-compensated). (b) CV characteristics of Ru/RGO electrodes in 2, 10, and 20 mmol FF and 1 M aqueous KOH as the electrolyte with a scan rate of 20 mV s^–1^. (c) ECO of FF and yield of 2-FA and 5-HFA over RGO, Ru, and Ru/RGO electrodes. Reaction conditions: 20 mmol FF, electrolyte 1 M KOH, optimum reaction time of 120 min, and potential of 0.8 V vs Ag/AgCl. (d) Various FF concentrations (5–30 mmol) vs ECO of FF and yield of 2-FA and 5-HFA over Ru/RGO electrodes. Reaction conditions: electrolyte 1 M KOH, optimum reaction time of 120 min, and anode potential of 0.8 V. (e) Various anode potentials (0.6–1.4 V vs Ag/AgCl) vs ECO of FF and yield of 2-FA and 5-HFA over Ru/RGO electrodes. Reaction conditions: 25 mmol FF; electrolyte, 1 M KOH; and optimum reaction time of 120 min. (f) Various electrolyte concentrations (0.1–2.0 M KOH) vs ECO of FF and yield of 2-FA and 5-HFA over Ru/RGO electrodes. Reaction conditions: 25 mmol FF, anode potential of 0.8 V vs Ag/AgCl, and optimum reaction time of 120 min. The electrochemical H-cell and symmetric Ru/RGO electrode systems were used for conducting these experiments.

The ECO of FF on the Ru/RGO anode was first investigated by CV in a three-electrode system consisting of Ru/RGO as the working electrode and Ag/AgCl and Pt as the reference and counter electrodes, respectively. The CV experiment was conducted over Ru and Ru/RGO at a scan rate of 20 mV s^–1^ with and without (bare) furfural present in 1 M aqueous KOH as the electrolyte, and the results are depicted in Figure 5a. In the bare CV run, the Ru/RGO electrode did not exhibit any notable anodic peak current at 20 mV s^–1^ in 1 M KOH without the addition of FF. In the CV characteristic curve for the metallic Ru electrode in the presence of 20 mmol FF, a high anodic current response was obtained at *I*_pa_ = 10.2 mA and the oxidation potential of *E*_pa_ = 0.82 V vs Ag/AgCl, respectively. The anodic *E*_pa_ at 0.82 V vs Ag/AgCl was associated with the oxidation of FF into 2-FA and 5-HFA with 2e^–^/H^+^ and/or 4e^–^/H^+^ processes.^6,11^ In addition, a well-enhanced anodic peak current of *I*_pa_ = 18.5 mA at an oxidation potential of *E*_pa_ = 0.83 V was observed at the Ru/RGO electrode in the presence of 20 mmol FF, which could be attributed to the higher FF adsorption capability and electronic conductivity of the RGO. In particular, RGO nanosheets possessed a higher surface area for adsorbing FF molecules, which brought FF molecules closer to the metallic Ru catalytic sites, thus promoting FF oxidation over the Ru/RGO surfaces. Further studies have discussed the influence of FF concentration on the Ru/RGO electrocatalytic activity of the Ru/RGO nanocomposite at a scan rate of 20 mV s^–1^ in 1 M KOH aqueous electrolyte solution, as displayed in Figure 5b. As a result, *I*_pa_ increased to 3, 10.5, and 19.0 mA, while *E*_pa_ values of 7.8, 8.0, and 8.2 V corresponded to increases in the FF concentrations to 2, 10, and 20 mmol, respectively, over the Ru/RGO electrode. This revealed that increasing the FF concentration up to 20 mmol minimally impacted the electrode electrocatalytic activity. Hence, microwave-assisted Ru/RGO possessed a higher surface area and higher electrocatalytic activity toward the ECO of FF into oxygenated products.

CV characteristic studies confirmed that the as-prepared Ru/RGO could be beneficial as a high-performing anode for the ECO of FF under the optimized conditions. An H-cell was used for the ECO of FF into targeted oxygenated products, including 2-FA and 5-HFA, over different anodes.^6^ The H-cell consisted of anodic and cathodic chambers separated by a proton exchange membrane, and the chamber was filled with 1 M KOH as the aqueous electrolyte and 20 mmol FF as the anodic reactant for the ECO of FF. Figure 5c compares the ECO FF and product yields over RGO-, Ru-, and Ru/RGO-based anodes at 1.0 V vs Ag/AgCl of the anode potential in a 1 M KOH aqueous solution. The microwave-assisted RGO-based anode afforded 26% and 9% yields of 2-FA and 5-HFA, respectively, at ECO *X*_FF_ = 35%. The metallic Ru anode afforded 41.5% yield of 2-FA and 20.5% yield of 5-HFA at *X*_FF_ = 62% due to the presence of highly active metallic sites, thus potentially enhancing the ECO of FF under the same experimental procedure. Interestingly, the Ru/RGO anode showed a dramatic improvement in the ECO of FF with a 75% 2-FA yield and 15% 5-HFA yield at *X*_FF_ = 90%, and the carbon balance was approximately 99%. The fine dispersion of metallic Ru NPs exhibited more active catalytic sites, and the higher surface area of RGO enhanced the adsorption of FF molecules to facilitate the ECO of FF at the Ru/RGO surface. Furthermore, zeta potential analysis confirmed the adsorption ability of the RGO and Ru/RGO in terms of FF molecule adsorption. The pure FF, Ru, RGO, and Ru/RGO samples showed zeta potential values of −45, −0.2, −15, and −10 mV, respectively, while 20 mmol FF + Ru, 20 mmol FF + RGO, and 20 mmol FF + Ru/RGO exhibited zeta potentials of −44.5, −20, and −19 mV, respectively. These results clearly depicted that the FF molecules were preferably adsorbed on the RGO nanosheet surfaces via electrostatic interactions than on the Ru surfaces. The observations also confirmed that FF molecules were strongly physisorbed on RGO, and the active Ru NPs spilled over the RGO surface would easily enhance the FF ECO and the product yields.

The effect of FF concentration on the ECO of FF and product yields was determined over the Ru/RGO catalyst in the FF concentration range of 5–30 mmol at 1.0 V vs Ag/AgCl in 1 M KOH aqueous electrolyte solution, and the results are shown in Figure 5d. At a 5 mmol FF concentration, 44% 2-FA yield and 16% 5-HFA yield were estimated at *X*_FF_ = 60%. However, when the FF concentration increased from 5 to 25 mmol, the 2-FA yield increased from 43.5% to 77%, while the 5-HFA yield slightly increased from 16% to 17% at a maximum of *X*_FF_ = 94%. In addition, with an increase in FF concentration up to 30 mmol, *X*_FF_ and product yields were almost constant due to limited electrode area availability at the optimized reaction time. The influence of the applied cell voltage on the *X*_FF_ of ECO and the product yield over Ru/RGO was also studied, and the results are depicted in Figure 5e. The *X*_FF_ and product yields were estimated at various anodic potentials of 0.6–1.4 V vs Ag/AgCl. Initially, the *X*_FF_ increased from 70% to 94%, while the 2-FA yield and 5-HFA increased from 60% to 77% and 10% to 17%, respectively, when the cell potential increased from 0.6 to 0.8 V vs Ag/AgCl. The maximum 2-FA yield occurred at 0.8 V vs Ag/AgCl in 1 M KOH aqueous solution, which indicated that the 0.8 V vs Ag/AgCl cell potential easily accelerates the 2e^–^ process for the production of 2-FA over the Ru/RGO electrode.^11^ Furthermore, *X*_FF_ was slightly increased from 94% to 96% as the anodic potential was increased from 1.0 to 1.4 V vs Ag/AgCl, as depicted in Figure 5e. At the same anodic potential (1.0–1.4 V vs Ag/AgCl), the 5-HFA yield increased from 25% to 61%, while the 2-FA yield decreased from 70% to 35%, indicating that higher anodic potentials accelerated the 4e^–^ process toward the selective 5-HFA production. The Ru/RGO electrode recorded a maximum 5-HFA yield of 61% at 1.4 V vs Ag/AgCl for 120 min in 1.0 M KOH electrolyte as the reaction medium. This investigation revealed that the lower cell potential (0.8 V vs Ag/AgCl) was favored for the 2e^–^ process, and a higher potential (1.4 V vs Ag/AgCl) accelerated the 4e^–^ process for the tunable production of 2-FA and 5-HFA at the Ru/RGO anode surfaces.^11^ Additionally, the influence of electrolyte concentration on the *X*_FF_ and selective production of 2-FA at the Ru/RGO electrode was examined over 0.1–2.0 M KOH aqueous electrolyte.

Figure 5f shows that the *X*_FF_ and 2-FA and 5-HFA yields increased with an increase in the electrolyte concentration from 0.1 to 1.0 M KOH aqueous solution at 0.8 V vs Ag/AgCl with 25 mmol FF. At the 1.0 M KOH electrolyte concentration, the maximum 2-FA yield of 77% along with a 5-HFA yield of 17% occurred at *X*_FF_ = 94%. Further increasing the KOH electrolyte concentration up to 2.0 M accelerated the oxygen evolution reaction, decreasing *X*_FF_ to 89% the yields of 2-FA (70%) and 5-HFA (29%). Thus, FF was effectively electrooxidized (*X*_FF_ = 94%) into a high 2-FA yield (77%) with an FE of 81% over the Ru/RGO electrode at 0.8 V vs Ag/AgCl in 1.0 M KOH aqueous electrolyte solution with 25 mmol FF as the anodic reactant.

**Figure 6:**
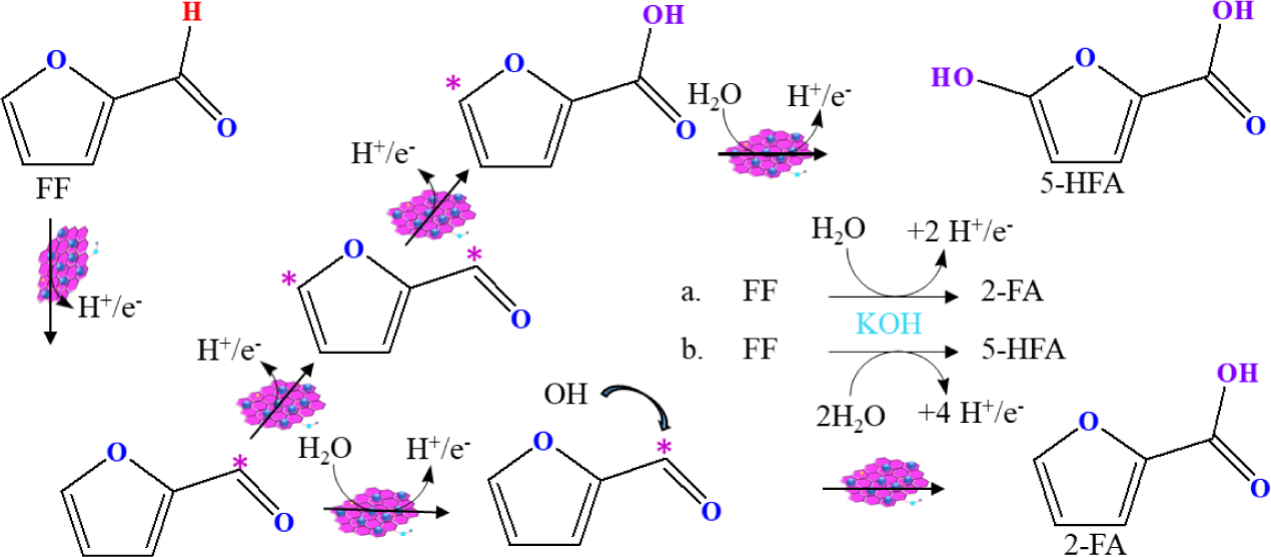
Plausible oxidation routes of FF to form the major products 2-FA and 5-HFA over Ru/RGO electrodes in 20 mmol FF and 1 M KOH as the electrolyte.

Figure 6 summarizes the proposed pathway for the ECO of FF in the selective formation of 2-FA and 5-HFA over Ru/RGO electrodes by sequential oxidation. This scheme was depicted on the basis of the ECO of FF and product yields over the Ru/RGO anode, as well as recently reported studies.^11^ Initially, FF molecules were attached to the RGO surfaces via electrostatic interactions, which came closer to the Ru catalytic sites in the anode. Metallic Ru catalytic sites effectively dehydrogenate the adsorbed FF molecules along with the discharge of H^+^ + e^–^, and then, the dehydrogenated FF combines with OH* to form 2-FA at the Ru/RGO anodic surface.^6^ In addition, the dehydrogenated FF molecules are transformed into 5-HFA at Ru/RGO via H-abstraction at the 5-position of the ring under a higher cell potential. This investigation clearly showed that FF was simply electrooxidized into 2-FA and 5-HFA along with plenty of H^+^ + e^–^ discharge.^10,11^ The ECO of FF to 2-FA and 5-HFA is a 2e^–^ and 4e^–^ process, respectively. Hence, the discharged +2H^+^/e^–^ and 4H^+^/e^–^ will potentially be utilized for the ECH of FF into FFA and 2-MF in the paired electrolyte cell.^8^

### 3.4 Paired Electrolyzer for Simultaneous ECH and ECO of Furfural

**Figure 7:**
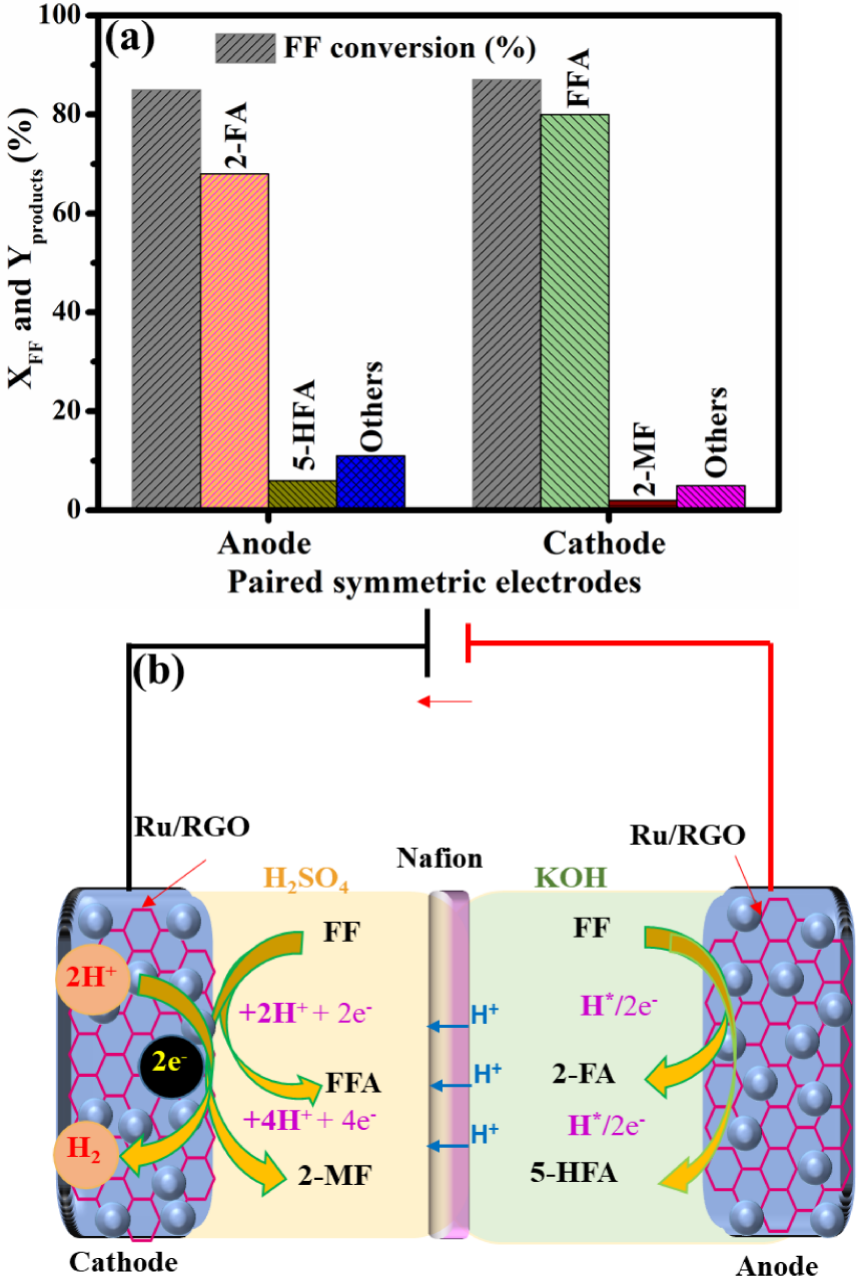
(a) Conversion of FF and yields of the desired products over paired symmetric electrodes with an anode and cathode. Reaction conditions: cathode potential of −1.25 V vs Ag/AgCl, cathode electrolyte of 2.0 M H_2_SO_4_, anode electrolyte of 1.0 M KOH, and a reaction time of 120 min. (b) Schematic illustration of the paired symmetric electrode system for the electrochemical hydrogenation and oxidation of FF and yields of the desired products. All electrochemical H-cells and Ru/RGO used as anodes and cathodes were used for these studies.

With the aforementioned results in hand, Ru/RGO was used as a common electrode for the paired electrode systems, simultaneously achieving both ECH and ECO of FF. Hence, a paired electrolyzer cell was constructed with two anodic and cathodic chambers separated by a proton exchange membrane. An anodic chamber was filled with 1.0 M KOH and 25 mmol FF, while the cathodic chamber was filled with 2.0 M H_2_SO_4_ with 20 mmol FF. The paired electrolyzer cell was operated with a controlled cell potential of −1.25 V. Figure 7a shows the individual *X*_FF_ and anodic and cathodic product yields in the paired electrolyzer. In the anodic chamber, 85% *X*_FF_ with 60% 2-FA and 14% 5-HFA yields along with 11% of other oxygenated products, including maleic acid (5%), 2(3*H*)-furanone (3%), and 5-hydroxyfuran-2(5*H*)-one (4%), were observed at Ru/RGO surfaces; this described the further oxidation of 2-FA and 5-HFA into 6e^–^ and 8e^–^ products at more elevated potentials.^7,10^ However, the yield of 2-FA and 5-HFA can be maximized by decreasing the cell potential and operating it in the range of −0.5 to −0.8 V. In the cathode, FF was electrochemically hydrogenated into FFA and 2-MF along with other hydrogenated compounds at the Ru/RGO cathode. As shown in Figure 7a, 91% 2-MF and 8% FFA yields along with 1% undesired hydrogenated products occurred at *X*_FF_ = 97% along with a carbon balance of 99%. The obtained paired electrolyzer results are very competitive with those obtained in the half-cell electrolyzer. Notably, the paired electrolyzer possessed a higher 2-MF yield of 91% than the 2-MF yield (88%) obtained from the cathodic half-cell electrolyzer. This result indicates that anodic H^+^ and e^–^ produced could have potentially participated in the ECH of FF, thus enhancing selective 2-MF formation with a high FE of 95%.^5^Figure 7b illustrates the mechanism of the paired electrolyzer in the simultaneous ECH and ECO of FF at a controlled cathodic potential of −1.25 V. During the ECO of FF, the oxygenated 2-FA and 5-HFA products are the preferred targeted compounds along with production of H^+^ and e^–^, which are key to producing the desired 2-MF in the cathodic chamber. In the anodic chamber, 2e^–^ (2-FA), 4e^–^ (5-HFA), 6e^–^ (maleic acid), and 8e^–^ (5-hydroxyfuran-2(5*H*)-one) were the major oxygenated products that discharged H^+^ and e^–^.^6,10,11^ Then, H^+^ reached the cathodic chamber through the proton exchange membrane, while e^–^ moved to the cathode by a potentiostat for FF ECH.^19^ The anodic and cathodic reaction products in the paired electrolyzer can be represented as follows:
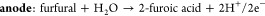
5
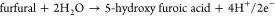
6
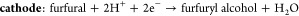
7
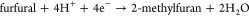
8

Notably, the ECO of FF promotes the ECH of FF toward selective 2-MF production in the paired electrolyzer. This intriguing electrochemical performance suggests that the paired electrode design using Ru/RGO is a promising and cost-effective material for the simultaneous reduction and oxidation of FF owing to its higher surface area, electrical conductivity, and higher active metallic catalytic sites toward sustainable liquid fuel production. Compared to half-cell electrolyzers, paired electrolyzers are highly efficient and technoeconomic devices for industrial applications.

## 4 Conclusion

This work focused on the development and characterization of a unique Ru/RGO nanocomposite electrocatalyst system whose salient features are as follows:

1. A microwave method was used to synthesize the Ru/RGO nanocomposite at 1400 W operating power within a short duration of 30 s.
2. HR-TEM and FE-SEM analysis revealed that the ultrasmall diameter of Ru NPs with average sizes of 3.5 nm was uniformly dispersed on the 2D RGO surfaces.
3. The microwave-synthesized Ru/RGO nanocatalyst possessed a higher surface area of 185 m^2^ g^–1^ and a total pore volume of 0.22 cm^3^ g^–1^, which was beneficial for the adsorption of FF compounds through electrostatic interactions and the ion transport properties.
4. CV characteristic studies revealed that Ru/RGO exhibited a higher FF electrocatalytic reduction at a lower cathodic potential of −1.25 V vs Ag/AgCl with a scan rate of 20 mV s^–1^ in 1.0 M H_2_SO_4_ as the electrolyte solution.
5. Additionally, CV characteristic studies clearly depicted that FF was efficiently electrooxidized at a scan rate of 20 mV s^–1^ with a lower anodic potential of 0.8 V vs Ag/AgCl in 1.0 M KOH as the electrolyte solution.

A paired electrochemical system was fabricated using Ru/RGO for the simultaneous ECH and ECO of furfural into valuable chemicals. The ECH of FF into FFA and 2-MF products was optimized by examining the catalyst effect, various FF concentrations (5–30 mmol), different applied cell potentials (−0.8 to −1.4 V vs Ag/AgCl), and electrolyte concentrations (0.1–2.0 M H_2_SO_4_), respectively. The cathodic half-cell possessed a higher yields of 2-MF (88%) and FFA (6%) along with *X*_FF_ (94%) for the Ru/RGO electrode, while the anodic half-cell studies established the ECO of FF into the selective 2-FA and 5-HFA products. Notably, 77% 2-FA yield with FE of 81% and 16% 5-HFA yield at *X*_FF_ = 94% occurred with abundant H^+^ and e^–^ discharged over the Ru/RGO anode. Interestingly, the discharged anodic H^+^ and e^–^ products could be utilized for ECH of FF into the desired 2-MF product under a controlled cathodic potential of −1.25 V. For this study, a paired electrolyzer was constructed with Ru/RGO as bifunctional electrodes for simultaneous ECH and ECO of FF. Under these conditions, FF was electrooxidized into 2e^–^ (2-FA), 4e^–^ (5-HFA), 6e^–^ (maleic acid), and 8e^–^ (5-hydroxyfuran-2(5H)-one) anodic products with H^+^ and e^–^, and the discharged H^+^ and e^–^ participated in the ECH of FF, leading to a higher 2-MF yield (91%) at *X*_FF_ = 97%. The paired electrolyzer mechanism was discussed on the basis of these results and the existing literature. In particular, the paired electrolyzer cell showed a higher 2-MF yield than a half-cell reaction, which is an economically feasible technique for sustainable liquid fuel production and further conversion of various organic compounds.

## Supporting Information Available

Scan rate dependence of the current densities in the CV curves of Ru and Ru/RGO electrodes and double-layer capacitance measurements to determine the electrochemically active surface areas of the Ru and Ru/RGO electrodes (PDF)
