# Supplementary material for: High-Grade Biofuel Synthesis from Paired Electrohydrogenation and Electrooxidation of Furfural Using Symmetric Ru/Reduced Graphene Oxide Electrodes
Source: ACS Appl Mater Interfaces. 2021 May 19;13(21):24643–53. doi: 10.1021/acsami.1c02231 (PMC8289174; doi:10.1021/acsami.1c02231)
Supplement: Supplementary file 1 — am1c02231_si_001.pdf [file am1c02231_si_001.pdf]

Supporting Information

High-Grade Biofuel Synthesis from Paired Electro-Hydrogenation and Electro-Oxidation of Furfural Using Symmetric Ru/Reduced Graphene Oxide Electrodes

G. Bharath,* Fawzi Banat

Department of Chemical Engineering, Khalifa University, P.O. Box 127788, Abu Dhabi, United Arab Emirates

Corresponding E-mails: Dr. G. Bharath sribharath7@gmail.com and fawzi.banat@ku.ac.ae

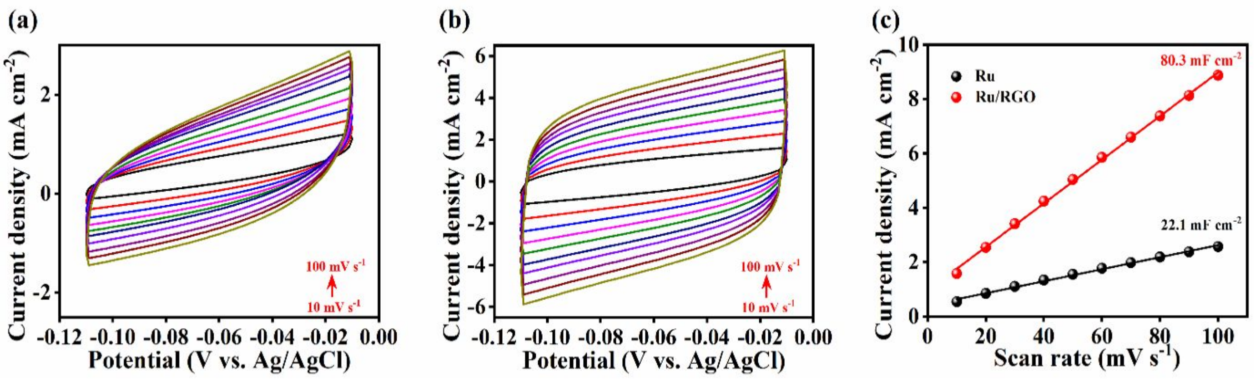

Figure S1. Scan rate dependence of the current densities in the CV curves of (a) Ru electrode, (b) Ru/RGO electrodes with scan different sweep rates from 10 to 100 mV s^{-1} at intervals of 10 mV s^{-1}, and (c) Double-layer capacitance measurements to determine the electrochemically active surface areas of the Ru and Ru/RGO electrodes.
